# Crosstalk Between Inflammation and Cellular Senescence in Osteoarthritis: Modulatory Actions of Olive Oil and Its Bioactive Compounds

**DOI:** 10.1155/mi/5347893

**Published:** 2026-06-09

**Authors:** Alessandro Medoro, Roberta Tardugno, Sergio Davinelli, Maria Lisa Clodoveo, Eugenio Luigi Iorio, Magda Tsolaki, Filomena Corbo, Giovanni Scapagnini

**Affiliations:** ^1^ Department of Medicine and Health Sciences “V. Tiberio”, University of Molise, Campobasso, Italy, unimol.it; ^2^ Department of Pharmacy-Drug Sciences, University of Bari “Aldo Moro”, Bari, Italy, uniba.it; ^3^ Italian Nutraceutical Society (SINut), Bologna, Italy; ^4^ Interdisciplinary Department of Medicine, School of Medicine, University of Bari “Aldo Moro”, Bari, Italy, uniba.it; ^5^ Program of Post-Graduation in Health Science, Federal University of Uberlandia, Uberlandia, Brazil, ufu.br; ^6^ 1st Department of Neurology, Medical School, Aristotle University of Thessaloniki, Thessaloniki, Greece, auth.gr; ^7^ Laboratory of Neurodegenerative Diseases, CIRI, Aristotle University of Thessaloniki, Thessaloniki, Greece, auth.gr

## Abstract

Osteoarthritis is a degenerative joint disorder characterized by progressive cartilage degradation, subchondral bone alterations, and persistent low‐grade inflammation. Recent findings have identified a strong interplay between inflammation and cellular senescence in osteoarthritis. Chronic inflammatory signals promote the accumulation of senescent cells, while senescent cells amplify inflammatory pathways through their senescence‐associated secretory phenotype (SASP). This bidirectional loop accelerates tissue damage by perpetuating oxidative stress, matrix degradation, and the release of proinflammatory mediators that reinforce the senescence process. Likewise, the biological activities of olive oil and its bioactive compounds, including monounsaturated and polyunsaturated fatty acids (PUFA), phenolic compounds (mainly ligstroside, oleocanthal, oleuropein, and hydroxytyrosol), squalene, phytosterols, vitamins (particularly vitamin E), and carotenoids, have attracted increased attention. These compounds may synergistically exert their effects through several interrelated mechanisms that influence both inflammatory and senescence pathways. They may modulate key signaling cascades, such as nuclear factor‐*κ*B (NF‐*κ*B), mitogen‐activated protein kinases (MAPKs), and Toll‐like receptors (TLRs), that drive the release of proinflammatory cytokines. Moreover, by attenuating SASP, olive oil compounds can potentially attenuate the vicious cycle between inflammation and senescence, slowing cartilage degradation and preserving joint function. Here, we synthesize current findings on the molecular mechanisms and clinical implications of bioactive compounds from olive oil, emphasizing their role in modulating both inflammation and senescence during osteoarthritis.

## 1. Introduction

Osteoarthritis is the most common chronic, progressive joint disorder and is characterized by the degeneration of articular cartilage, alterations in subchondral bone, synovial inflammation, and changes in periarticular tissues [1]. Historically classified as an aseptic “non‐inflammatory” arthropathy, recent evidence has redefined osteoarthritis as a multifactorial disease where chronic, low‐grade inflammation plays a pivotal role in both its onset and progression. Joint injury, repetitive mechanical stress, and aging contribute to a complex inflammatory mechanism with the release of mediators that collectively lead to extracellular matrix (ECM) degradation and joint tissue damage. Alongside these inflammatory events, cellular senescence has emerged as a key factor in osteoarthritis pathology. Senescence is characterized by irreversible cell cycle arrest and a distinct secretory profile known as the senescence‐associated secretory phenotype (SASP), which includes numerous proinflammatory cytokines, chemokines, growth factors, and proteases. In osteoarthritic joints, senescent chondrocytes accumulate and worsen tissue degeneration by releasing SASP factors that aggravate local inflammation and matrix breakdown. This interplay creates a vicious cycle between chronic inflammation and cellular senescence [2].

Given the complex network of inflammatory and senescence‐driven pathways in osteoarthritis, there is growing interest in therapeutic strategies that target both processes simultaneously. Among the most promising candidates, bioactive compounds from olive oil, a central component of the Mediterranean diet, may become potential candidates as senotherapeutic agents to alleviate inflammation and SASP in osteoarthritis. Olive oil is abundant in oleic acid and polyphenols such as hydroxytyrosol, ligstroside, oleocanthal, and oleuropein, which exhibit potent anti‐inflammatory, antioxidant, and senotherapeutic properties. Preclinical studies have demonstrated that these compounds can inhibit key inflammatory pathways, including nuclear factor *κ*‐B (NF‐*κ*B) and mitogen‐activated protein kinase (MAPK) signaling, thereby reducing the expression of proinflammatory mediators and enzymes that degrade cartilage [3]. Furthermore, olive oil bioactive compounds have been shown to counteract the SASP, diminishing the deleterious paracrine effects of senescent cells on joint tissues [4].

Although still limited, preliminary clinical studies have shown promising evidence for using olive oil–based formulations in managing osteoarthritis. For example, both topical applications and oral supplementation with olive oil have been linked to improvements in joint pain, reduced inflammation, and better mobility in small groups of patients [5–8].

In this narrative review, we provide a detailed overview of the molecular mechanisms underlying the interactions between inflammation and cellular senescence in osteoarthritis. We also examine the emerging evidence regarding the anti‐inflammatory and senotherapeutic effects of olive oil bioactive compounds. By combining findings from preclinical studies with preliminary clinical data, we aim to highlight the potential of olive oil in slowing the progression of osteoarthritis, describing and analyzing signaling pathways involved in osteoarthritis onset and progression, and offering an updated overview of current evidence on this emerging topic.

## 2. Inflammation and Cellular Senescence in Osteoarthritis

Osteoarthritis, although historically classified as an aseptic “non‐inflammatory” arthropathy, involves a multifaceted inflammatory response that affects not only the cartilage but also the subchondral bone, synovial membrane, and infrapatellar fat pads [9, 10]. The cell defense system initiates inflammation as an adaptive response to harmful stimuli, including infections and tissue damage [11]. This inflammatory response is tightly regulated and highly specific. Detection of harmful stimuli occurs via pattern recognition receptors (PRRs) on the cell surface, which recognize pathogen‐associated molecular patterns (PAMPs) and danger‐associated molecular patterns (DAMPs) released during cellular or tissue injury. The activation of PRRs triggers several intracellular signaling pathways, including NF‐*κ*B and MAPK pathways [12]. These signaling cascades stimulate inflammatory cells, such as macrophages and adipocytes, to release inflammatory mediators, including cytokines. At the same time, a coordinated network of diverse cell types recruits activated macrophages, monocytes, and other immune cells to sites of tissue injury or infection [13].

Cytokines and chemokines, including the proinflammatory interleukins (IL)‐6, IL‐8, IL‐15, and IL‐33, are secreted by the aforementioned cells, with their secretion increasing in parallel with the expression of DAMPs. Inflammation‐triggering mediators, such as IL‐1*β* and tumor necrosis factor *α* (TNF‐*α*), are released during the early stages of osteoarthritis [2, 14]. TNF‐*α* interacts with TNF receptor 1 (TNFR1) and TNF receptor 2 (TNFR2), thereby activating downstream signaling pathways. Both receptors are expressed in the synovial membrane, with TNFR1 strongly promoting proinflammatory responses, while TNFR2 exhibits context‐dependent roles, exerting either proinflammatory or anti‐inflammatory effects based on the pathological condition [15].

These proinflammatory mediators stimulate the accumulation of reactive oxygen species (ROS) and the production of significant amounts of nitric oxide (NO) via inducible nitric oxide synthase (iNOS), which subsequently enhances the synthesis of prostaglandin E2 (PGE2) and cyclooxygenase‐2 (COX2). Concurrently, PGE2 facilitates the production of matrix metalloproteinase 13 (MMP13), a key enzyme responsible for collagen degradation [16].

**Figure 1:**
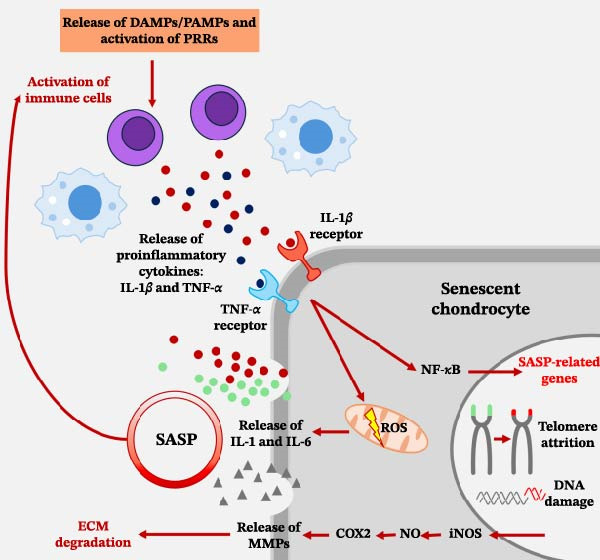
Interplay between inflammation and cellular senescence in osteoarthritis progression.

The pathogenesis of osteoarthritis arises from the interplay between inflammation and senescence, with these two processes exerting reciprocal influence (Figure 1). Cellular senescence can be defined as a form of permanent growth arrest triggered by various stressors, including telomere attrition, oncogene activation, and DNA damage. Senescent cells exhibit distinct features such as morphological alterations (e.g., enlarged, flattened shape), expression of specific markers (e.g., p16^INK4a^, p21^Cip1/Waf1^), and activation of peculiar pathways (e.g., NF‐*κ*B). A hallmark of this state is the SASP, which is characterized by the release of numerous proinflammatory mediators (e.g., IL‐1, IL‐6, IL‐7, IL‐8, IL‐18, TNF‐*α*), growth factors, and metalloproteinases (MMP1 and MMP10). The SASP not only modulates the local tissue microenvironment but also sustains chronic inflammation and promotes tissue remodeling, thereby contributing to disease progression [17, 18].

Chronic low‐grade inflammation promotes senescence through multiple pathways, including hyperactivation of the immune system (immunosenescence), tissue degeneration, and impaired stem cell functionality [19]. Inflammation often precedes the development of senescence and proinflammatory cytokines activate the inflammatory response in surrounding cells and further sustain SASP; the prolonged presence of SASP exacerbates chronic inflammatory responses, creating a self‐perpetuating feedback loop. Senescent cells secrete IL and chemokines, which act on neighboring cells in a paracrine manner. SASP factors act on neighboring cells to induce a late senescent state among young and early senescent cells, promoting a senescent microenvironment [2].

Proinflammatory mediators such as IL‐1*β* and TNF‐*α* typically do not act in isolation when driving senescence; they also trigger ROS accumulation, synergistically expediting cell damage. Indeed, Yagi et al. [20] identified ROS as a key driver of inflammation‐induced senescence.

Chondrocytes are the primary resident cells in articular cartilage and are essential for maintaining its structural integrity and functional properties. These cells play a pivotal role in cartilage homeostasis and are also central to the development and progression of osteoarthritis. Among the various cell types in the joint, chondrocytes are the most significantly affected by the senescence process. Despite their inherently poor self‐renewal capacity, chondrocytes exhibit some limited proliferative potential under specific conditions, such as during early tissue repair [21]. However, this capacity is insufficient to counteract the progressive degenerative changes observed in osteoarthritis [22].

Senescence in chondrocytes is a critical driver of pathological changes within the joint. Although the causes are not entirely understood, evidence indicates that chondrocyte senescence can be induced by various factors, including nutritional deficiencies, hypoxia, ROS, DNA damage, protein aggregation, organelle dysfunction, or intracellular pathogens. The senescence in chondrocytes is characterized by telomere attrition, decline in mitotic activity, cell cycle arrest, and decreased functional capacity [23]. Senescent chondrocytes contribute to osteoarthritis progression primarily through intercellular communication mechanisms, including chronic low‐grade inflammation, a process referred to as “inflammsenescence” [2]. The interaction between senescence, inflammation, and metabolic dysregulation is supported by studies demonstrating a metabolic shift in chondrocytes during inflammatory states. NF‐*κ*B activation reprograms cellular glycolysis, with increased lactate dehydrogenase A expression driving ROS‐induced catabolism. NF‐*κ*B inhibitor zeta (I*κ*B‐ζ), a downstream mediator of NF‐*κ*B, has been shown to regulate the NF‐*κ*B ligand (RANKL), inflammation, and catabolism, reprogramming chondrocytes into an inflammatory phenotype [24–26]. Therefore, several studies have indicated that the propagation of senescence is largely driven by the development of SASP. Through SASP‐associated intercellular communication, senescent chondrocytes facilitate the spread of senescence within joint tissues inducing pathological biochemical alterations in synoviocytes, osteoblasts, and immune cells, exacerbating the degenerative milieu of the joint. Moreover, SASP and defective or reduced chondrocyte capability contribute to a cascade of detrimental processes, including ECM degradation due to the expression of ECM‐degrading MMP1 and MMP13, increased oxidative stress, and heightened inflammatory signaling, which are also results of chondrocyte loss caused by chondrocyte apoptosis and necrosis. These alterations compromise the structural and functional integrity of cartilage and promote a feedback loop of cellular dysfunction. This feedback loop further amplifies tissue damage, leading to osteoarthritis onset and progression [27].

Among the different mediators implicated in the pathogenesis of osteoarthritis, several growth factors contribute to osteoarthritis progression through their influence on chondrocyte senescence, inflammation, and cartilage and bone remodeling. Vascular endothelial growth factor (VEGF) plays a particularly prominent role by linking angiogenesis, inflammation, and structural remodeling within the joint [28]. Aberrant VEGF expression is consistently observed in articular cartilage, synovium, synovial fluid, subchondral bone, and serum of patients with advanced disease, where its levels correlate with both structural damage and pain intensity [28–31]. At the cellular level, VEGF may stimulate chondrocyte SASP, inducing matrix metalloproteinases (MMP‐1, MMP‐3, MMP‐13), NO, and proinflammatory cytokines, while suppressing extracellular matrix synthesis, particularly aggrecan and type II collagen, contributing to cartilage degradation, osteophyte formation, bone sclerosis, and synovial hyperplasia [28, 32–36].

In addition to VEGF, nerve growth factor (NGF) plays a multifaceted role in osteoarthritis, acting as a mediator of inflammation and pain and as a modulator of cartilage metabolism. While anti‐NGF antibodies have shown remarkable efficacy in reducing osteoarthritis‐related pain, their clinical use has been limited by the occurrence of rapidly progressive osteoarthritis in a subset of treated patients, suggesting that NGF signaling may also participate in cartilage inflammation and integrity. Increased NGF expression has been consistently observed in degenerated cartilage, where mechanical stress, inflammatory cytokines, and transforming growth factor *β* (TGF‐*β*) stimulate its production by chondrocytes [37].

## 3. Chemical Composition of Olive Oil

Olives and olive oil are traditional foods with thousands of years of history; in fact, they can be recognized as components of human civilization. Since the Neolithic era, people have utilized drupes and their oil for a variety of purposes, primarily as food, medicine, and cosmetics. Today, olive and olive oil are widely consumed worldwide and are key ingredients of the Mediterranean diet. Indeed, the nutritional and beneficial properties of olive and olive oil are strictly related to the chemical composition [38, 39].

The olive tree (*Olea europaea* L.) belongs to the *Oleaceae* family, which comprises ~30 genera and 600 species capable of adapting to a wide range of climates, both wild and cultivated [38, 40]. Phytochemical studies on the composition of *O. europaea* have revealed diverse and unique secondary metabolites in different parts of the tree. Focusing on the olive fruits (drupes), the presence of lipids, proteins, and minerals in their composition highlights their value as a significant source of nutrients for human consumption [38]. From the olive drupes, olive oil is obtained by mechanical processes, according to the European Commission regulation (EC, 2022/2104). The olive oils are chemically complex mixtures constituted by a main lipidic portion, namely the saponifiable fraction (98%–99% of the olive oil’s weight), and a minor fraction (<2% of the olive oil’s weight) containing chemical compounds belonging to a wide range of chemical classes, namely the unsaponifiable fraction [38–40].

### 3.1. Saponifiable Fraction: Saturated and Unsaturated Tri‐, Di‐Monoglycerides, and Free Fatty Acids

The saponifiable fraction contains tri‐, di‐, and monoglycerides and free fatty acids, essential lipid constituents’ indicative of the quality and genuineness of olive oils [41–43]. The fatty acids can be divided into saturated and unsaturated fatty acids. Saturated fatty acids do not contain carbon–carbon double bonds in their chain structure, while unsaturated fatty acids contain one double bond (monounsaturated fatty acids [MUFA]) or more double bonds (polyunsaturated fatty acids [PUFA]) respectively in their hydrocarbon chain structure. The double bonds of PUFA in the *cis* configuration contribute to their rounded molecular shape, and the polyunsaturated structure of the methylene bridge facilitates molecular rotation by improving its flexibility. The “rounded” shape and flexibility of PUFA lower the melting point and increase the fluidity of the lipid structure. Among the PUFA, *α*‐linolenic acid (ALA, *ω*‐3) and linoleic acid (LA, *ω*‐6) are classified as essential fatty acids because the human body cannot synthesize them, and they must be obtained through the diet [43]. MUFA/SFA ratio and *ω*6/*ω*3 ratio are commonly utilized to elucidate the role of dietary PUFA in the pathogenesis of cardiovascular diseases, cancer, and inflammatory and autoimmune disorders. From a basic perspective, a markedly high *ω*6/*ω*3 ratio is often regarded as negative to human health, whereas a ratio approaching 1 is considered to confer protective effects against these conditions [42].

As regards olive oil fatty acid composition, the average concentration reported by European Commission regulation (EC, 2022/2104) is constituted as follows in decreasing order: oleic acid (C18:1 n‐9, 55%–85%), linoleic acid (C18:2 n‐6, 2.5%–21%), palmitic acid (C16:0, 7%–20%), stearic acid (C18:0, 0.5%–5.0%), palmitoleic acid (C16:1, 0.3%–3.5%), and linolenic acid (C18 : 3, ≤1.00%) [40].

Omega‐3 fatty acids present in olive oils are examples of lipids that positively influence health; indeed, two health claims applicable to olive oil approved by the European Food Safety Authority (EFSA) refer to the high presence of oleic acid and the health benefits of replacing saturated fatty acids with unsaturated ones [44–46]. It has been established that several factors such as tree variety, geographic area, climatic conditions including light, temperature, and water availability, cultivation conditions, the stage of ripeness of the fruit and postharvest treatments as well as the characteristic genes involved in the biosynthesis of monounsaturated—and polyunsaturated—fatty acids, influence the FA composition of olive oils [40, 47].

### 3.2. Unsaponifiable Fraction: Phenolics, Alcohols, Sterols, Hydrocarbons, Vitamins, Pigments, Phytosterols, and Minerals

**Figure 2:**
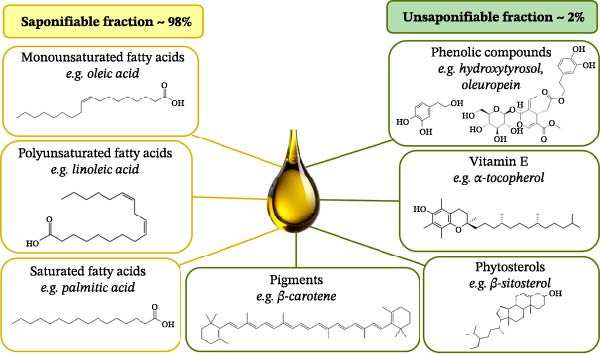
Chemical composition of olive oil.

The unsaponifiable fraction contains more than 200 different compounds, including phenolics, aliphatic and triterpenic alcohols, sterols, hydrocarbons (squalene), vitamins (tocopherols), pigments (*β*‐carotene), phytosterols, and minerals (Figure 2) [42, 48, 49]. Despite modest quantities, these minor components have a key role in influencing the nutritional value and health benefits of olive oils [50, 51]. Numerous studies have investigated their biological properties and beneficial effects on human health being phenolic compounds present in olive oil strong antioxidants and ROS scavengers [50–52]. Moreover, the phenolic fraction of olive oils also influences the sensory characteristics and plays a key role in their stability against oxidative factors [42, 52].

#### 3.2.1. Phenolic Compounds: Flavonoids, Lignans, Phenolic Alcohols, and Secoiridoids

The phenolic compounds identified and quantified in olive oil can be classified into: secoiridoids, phenolic alcohols, lignans, and flavonoids.

##### 3.2.1.1. Secoiridoids

Secoiridoids are secondary metabolites uniquely found in the *Oleaceae* family of plants, as well as a few other species. These compounds are a type of iridoid; in particular, they are derivatives of cyclopentane monoterpenes [53]. Their biosynthesis involves the condensation of tyrosol or hydroxytyrosol (from phenylpropanoid metabolism) with intermediates produced by the plant, resulting in the formation of glycosylated forms of oleuropein and ligstroside through the secoiridoid biosynthetic pathway [54]. During the process of olive oil extraction, crushing the olives releases glycosidases that break down the sugar component of oleuropein and ligstroside, yielding their aglycones. These aglycones undergo demethylation and spontaneous decarboxylation, producing the dialdehyde compounds oleocanthal and oleacein. As a result, olive oil is an exclusive source of these substances. The most prevalent secoiridoids in olive oils include the mono‐aldehydic forms of ligstroside aglycones (*p*‐HPEA‐EA) and oleuropein (3,4‐DHPEA‐EA), as well as the di‐aldehydic forms of their decarboxymethylated derivatives, oleocanthal (*p*‐HPEA‐EDA) and oleacein (3,4‐DHPEA‐EDA). Oleocanthal is found in olive oil but not in olives [54, 55]. The total content of secoiridoids varies according to cultivar, pedoclimatic conditions, and olive oil production processes; it can reach 1000 mg/kg [56].

##### 3.2.1.2. Phenolic Alcohols

The phenolic alcohols include many phenolic acids (vanillic, coumaric, caffeic, protocatechuic, *p*‐hydroxybenzoic, ferulic acid), as well as tyrosol and hydroxytyrosol, which are the most representative and abundant phenolic compounds in olive oil along with oleuropein. In addition, when esterified with elenolic acid, they form secoiridoids. Tyrosol (*p*‐hydroxyphenethyl ethanol, *p*‐HPEA) and hydroxytyrosol (3,4‐dihydroxyphenyl ethyl ethanol, 3,4‐DHPEA) have the same chemical structure except that hydroxytyrosol has an extra hydroxy group at the *meta* position. Typical values range from 100 to 300 mg/kg. The variability of olive oils can reach a total phenolic level below 100 mg/kg but also above 900 mg/kg, showing significant differences between different varieties and conditions as previously mentioned for other chemical classes [55, 57–59].

##### 3.2.1.3. Lignans

Lignans are generally considered phenolic compounds with a structure resembling that of steroids and are often referred to as phytoestrogens. The natural lignans present in olives and olive oils include pinoresinol and 1‐acetoxypinoresinol. Pinoresinol (C_20_H_22_O_6_) and 1‐acetoxypinoresinol (C_22_H_24_O_8_) are characterized by a furofuran ring system linked with two phenol groups in their chemical structure. Each phenol group consists of an aromatic ring (phenyl or benzene ring) attached to a hydroxyl group (OH). The phenol groups are associated with various health benefits in humans, including antioxidant and/or anti‐inflammatory effects. Lignans having a chemical structure similar to estrogen, in addition, could act as hormonal modulators in various pathologies [58, 59]. In plants, lignans are biosynthesized along the phenylpropanoid pathway, resulting in the biosynthesis of lignin, a plant‐based cell wall polymer [60]. While (+)‐pinoresinol has been identified in other plant species, 1‐acetoxypinoresinol is uniquely found in olives [59, 61]. Pinoresinol ranging from 0.24 to 0.14 mg/100 g exhibits considerable variability and demonstrates greater stability compared to other phenolic compounds under various conditions of malaxation, olive stoning, fruit maturity, or storage duration [58, 59]. After digestion by the intestinal microbiota, lignans are transformed into more active and bioavailable metabolites known as enterolignans, enterolactone, and enterodiol studied for their cardiovascular health benefits and preventive nutrition [62].

##### 3.2.1.4. Flavonoids

The flavonoids commonly found include apigenin and luteolin and their flavone glycosides (luteolin‐7‐O‐glucoside, apigenin‐7‐O‐glucoside [52]. These flavones have the widest distribution in plants and are extensively studied phenolics [63, 64]. Apigenin and luteolin are flavonoids within the flavone subclass, sharing a C6‐C3‐C6 core structure but differing in hydroxyl group positioning. Both compounds present a 15‐carbon skeleton that contains two benzene rings and a heterocyclic pyrone ring. Apigenin has three hydroxyl groups on the benzene rings, while luteolin has four hydroxyl groups on the benzene rings, which contributes to its higher polarity and biological activities [40, 57]. In addition to these, trace amounts of eriodictyol and naringenin, flavonoids that are rare in cultivated olive oils, have been observed in some olive oils from wild species [40, 57].

#### 3.2.2. Hydrocarbons

Olive oil is also among the richest food sources of squalene, containing up to 0.7% by weight. Squalene (C_30_H_50_) is the most representative hydrocarbon present in the unsaponifiable fraction of olive oil and a relevant qualitative marker [42, 65]. Its formula consists of six isoprene units connected in a head‐tail manner, forming a linear chain with six double bonds conjugated 1,4 with *trans* configurations. Due to this structure, squalene is highly susceptible to oxidation and can develop a fish odor if exposed to air for prolonged periods. It is a natural triterpene that has attracted attention for its potential health benefits and its widespread presence in both plant and animal sources. Squalene biosynthesis plays a key role in the metabolic pathway leading to sterol production, including cholesterol in animals and phytosterols in plants. It can be cyclized to form bicyclic, tetracyclic, and pentacyclic triterpenoid structures [46].

#### 3.2.3. Vitamins

In nature, vitamin E is present as a mixture of at least 8 compounds, including *α*‐, *β*‐, *δ*‐, and *γ*‐tocopherol and *α*‐, *β*‐, *δ*‐, and *γ*‐tocotrienol. The chemical structure is composed of a chromanol ring and a long tail of hydrocarbons. Tocopherols are vitamin E‐methylated forms of the chromanol ring and the main constituents of the unsaponifiable olive oil fraction. *α*‐tocopherol is the major tocopherol, representing 96% of the total content with a varying concentration between 80 and 500 mg/kg of olive oil (cultivars, agronomic conditions, and olive oil production influence the quantities [40, 66]. *α*‐tocopherol is followed by *β*‐tocopherol and *γ*‐tocopherol, while *δ*‐tocopherol is generally present in the lowest quantities both in cultivated and wild olives [40]. Vitamin E is well known for its protective action against the formation of ROS and lipid peroxides, which helps to preserve the quality and shelf life of olive oils [46].

#### 3.2.4. Pigments

Pigments are chemical compounds responsible for the characteristic green‐yellow color of olive oils present in the unsaponifiable fraction. Due to the lipophilic nature of olive oil, only chlorophylls and carotenoid pigments are transferred from the drupes to the oil phase. Carotenoids including *β*‐carotene, lutein, violaxanthin, neoxanthin, and other xanthophylls along with chlorophyll derivatives, including chlorophylls A and B, and pheophytins A and B, are the most representative pigments in olive oils [67]. Freshly pressed oils’ green color is due to a higher chlorophyll content than carotenoids. When the ratio of these compounds is close to 1, the color changes to yellowish. Chlorophylls are converted to pheophytins which retain their color. These compounds can be further transformed into colorless pyropheophytins and the degradation of carotenoids leads to colorless products in olive oils [40]. The major “yellow” pigments of virgin olive oils are lutein and *β*‐carotene [68].

The basic structure of a carotenoid consists of eight isoprene units containing conjugated double bonds (polyenes) and sometimes terminating in rings at one end. Carotenoids are classified into two main groups: hydrocarbons (carotenoids) and oxygenated derivatives (xanthophylls). Carotenoids such as *β*‐carotene have two six‐element rings on each side of the molecule and contain a *β*‐ionone ring that can act as provitamin A and antioxidants, while lutein does not possess provitamin A activity [69]. Carotenes improve their bioavailability when consumed together with long‐chain and unsaturated fatty acids [70].

Chlorophylls are based on a porphyrinic structure, comprising four pyrrole rings (*C*_4_H_4_NH) that are coordinated by a magnesium ion in the central position with a long hydrophobic alkyl chain attached to it. Chlorophylls and their derivatives have shown important health‐promoting functions, showing antioxidant and anti‐inflammatory activities. Among chlorophylls, pheophytin is found in major amounts in olive oils [71].

#### 3.2.5. Phytosterols

Plant sterols or phytosterols are lipid compounds produced by following the isoprenoid biosynthetic pathway. The basic structure of plant sterols resembles that of cholesterol, the skeleton consists of four condensed nonaromatic rings, of which three rings with six carbon atoms and one ring with five carbon atoms; a hydroxyl group at C3 in ring A; a double bond at C5/C7 in ring B; and a side alkyl chain at C17 in ring D. Sterols are present in the unsaponified fraction of olive oils as free and bound forms. They are important olive oil quali‐quantitative markers and possess health‐beneficial properties on human health. The most representative sterols (4‐desmethylsterols) in olives and olive oils are *β*‐sitosterol (75%–90% of total sterols), *Δ*5‐avenasterol (5%–20%), campesterol (1%–4%), and stigmasterol (0.5%–2%), although deviations in oils of some cultivars were also recorded. Less abundant sterols in olive oil are clerosterol, sitostanol, 24‐methylene‐cholesterol, *Δ*7‐avenasterol, *Δ*7‐campesterol, *Δ*5,24‐stigmastadienol, *Δ*7‐stigmastenol, and campestanol, and less commonly occurring sterols are *Δ*5,23‐stigmastadienol, cholesterol, and brassicasterol. erythrodiol and uvaol occur mainly below 4% of total sterols [72]. There is a large variability in the total sterol content among different olive oils, due to variety, geographical origin, harvest date, and other factors [40].

#### 3.2.6. Olive Oil Composition vs. Other Edible Oils

The lipid and phenolic composition of olive oil presents significant differences from other edible oils. Focusing on the fatty acid profile, olive oil is characterized by a high concentration of oleic acid, typically ranging from 55% to 83% of the total fatty acid content. This profile differs from that of higher saturated fatty acid content, primarily palmitic acid, found in other fruit‐derived oils, such as palm or pequi. While avocado oil shows a similar monounsaturated fatty acid percentage, olive oil maintains a distinct MUFA/SFA ratio compared to coconut or palm‐derived lipid fractions. Furthermore, in comparison to seed and nut oils (e.g., sunflower, soybean, and flaxseed), olive oil has lower levels of polyunsaturated fatty acids, specifically linoleic and *α*‐linolenic acids, which support its oxidative stability [42].

A chemotaxonomically relevant difference is based on the polar phenolic fraction. Secoiridoids, the main iridoids found only in the *Oleaceae* family, are biosynthesized by the mevalonate pathway from deoxyloganic acid. The connection of secoiridoids to the shikimate pathway is provided by two simple phenols, tyrosol and hydroxytyrosol, synthesized in the phenylpropanoid pathway. Indeed, while seed oils are characterized by high tocopherol levels, they lack these specific olive‐derived metabolites; conversely, olive oil contains a specific phytocomplex rich in secoiridoids, including oleuropein and ligstroside aglycones, as well as their hydrolytic derivatives hydroxytyrosol and tyrosol [54, 55]. These compounds are largely absent in fruit oils such as avocado or palm. Although certain nut oils (e.g., walnut or Brazil nut) may contain other specific lignans or flavonoids, the concentration and structural diversity of secoiridoids in olive oil define its characteristic phytochemical fingerprint. The presence of these olive‐specific secondary metabolites, combined with the aforementioned fatty acid profile, differentiates olive oil from other botanical lipid sources, providing the chemical basis for its nutritional and biological relevance [41, 42, 73].

#### 3.2.7. Bioavailability and Pharmacokinetic Properties

To understand the biological effects of olive oil chemical compounds, it is important to consider their absorption, distribution, metabolism, and excretion (ADME) properties and pharmacokinetics/pharmacodynamics, which determine the behavior and efficacy of bioactive compounds as well as food ingredients capable of acting on target tissues [73, 74]. Briefly, the triglycerides in the saponifiable fraction of olive oil undergo sequential hydrolysis in the stomach through gastric lipase, continuing in the duodenum with pancreatic lipase and bile secretion. This process produces diglycerides, monoglycerides, and free fatty acids that facilitate emulsification and enhance intestinal absorption of both major lipids and minor bioactive components of the unsaponifiable fraction, such as phenols [75]. Indeed, the lipid environment improves the solubilization and bioaccessibility of polyphenolic compounds [74, 76]. Focusing on main phenolics, hydroxytyrosol and tyrosol are present in olive oil both in free form and released from the related secoiridoids as oleuropein and ligstroside aglycones, whose ester bonds and secoiridoid ring opening chemically generate hydroxytyrosol and tyrosol [74, 77]. Further contributions to hydroxytyrosol and tyrosol amounts come from endogenous dopamine and tyramine metabolism [73, 74]. Hydroxytyrosol is absorbed in the small intestine primarily by passive diffusion, whereas oleuropein can also be transported by mediated absorption, with absorption efficiency depending on time and food matrix, and peak plasma concentrations typically reached within 30–60 min. Once absorbed, hydroxytyrosol is distributed to the kidneys, liver, muscles, and brain, with renal absorption being approximately tenfold more than that of other organs. Hydroxytyrosol binds to high‐density plasma lipoproteins, contributing to its antioxidant activity. Extended phase I and phase II metabolism generates mainly sulfated (e.g., hydroxytyrosol‐3‐O‐sulfate), glucuronidated, methylated (homovanillyl alcohol, homovanillic acid), and oxidized metabolites, with a relative proportion that depends on the dose (low doses promote glucuronidation; high doses promote sulfation). More lipophilic derivatives, such as hydroxytyrosol acetate and hydroxytyrosol acetate‐4^′^‐O‐sulfate, persist longer in plasma [73, 76, 77]. Free hydroxytyrosol is rarely detectable in plasma, indicating that biological effects are mediated primarily by these metabolites. In humans, excretion occurs mainly through the kidneys within about 6 h, while unabsorbed compounds or transformation products are eliminated in the feces [76, 78]. The gut microbiota contributes to the metabolic fate of phenols, generating additional metabolites with different activities and systemic duration [73].

Focusing on topical route, the high percentage of unsaturated fatty acids in olive oil, particularly oleic acid, is relevant to its dermal absorption profile. Indeed, it is known that unsaturated free fatty acids reversibly modulate the stratum corneum barrier by fluidizing intercellular lipids, thus improving skin permeation. In the epidermis, fatty acid metabolism transforms unsaturated fatty acids into related derivatives via the 15‐lipoxygenase pathway, suggesting that absorbed lipid compounds may undergo local biotransformation within the skin. Furthermore, transient barrier modification has been documented to enhance the permeation of both hydrophilic and lipophilic substances, indicating that this mechanism may also promote dermal uptake of several bioactive constituents present in the unsaponifiable fraction of olive oil, such as polyphenols, vitamin E, and other minor components [79, 80].

Overall, the described processes briefly summarize the oral and topical pathways of the main olive oil chemical bioactive constituents, which might reach peripheral tissues, including articular cartilage, possibly supporting their biological effects. Further investigations are still needed to deeply elucidate ADME and pharmacokinetics/pharmacodynamics of olive oil chemical compounds.

## 4. Anti‐inflammatory and Senotherapeutic Effects of Olive Oil and Derivatives in Osteoarthritis

Olive oil is rich in bioactive compounds, which have primarily demonstrated anti‐inflammatory and antisenescence properties, also in osteoarthritis animal models. For example, the intra‐articular extra‐virgin olive oil injection on mechanically induced rabbit knee osteoarthritis showed morphological, histological, and radiological improvement with potential cartilage restorative, senotherapeutic, and regenerative effects [81]. In the monoiodoacetate (MIA) rat model of osteoarthritis, a polyphenolic granular complex (100 mg/kg to 1 g/kg) obtained from olive pressing juice, enriched in total olive polyphenols, demonstrated a marked capacity to reduce osteoarticular pain. Acute administration attenuated responsiveness to acute inflammatory stimuli, mechanical hyperalgesia, and spontaneous pain. Moreover, a repeated daily treatment at 300 mg/kg completely abolished osteoarticular pain without evidence of tolerance on the antinociceptive effect [82]. In the same rat model of osteoarthritis, dietary extra‐virgin olive oil administered for 21 days reduced cartilage destruction and synovitis and preserved matrix proteoglycans, while lowering oxidative stress. Extra‐virgin olive oil‐fed animals showed reduced chondrocyte apoptosis, decreased proinflammatory oxidized low‐density lipoprotein (ox‐LDL), and lower total oxidative status and oxidative stress index, with a parallel increase in antioxidant capacity compared to untreated osteoarthritic rats [83].

Olive oil has also been investigated in combination with other compounds or treatments for potential synergistic effects on joint protection. In one study, Mével et al. administered olive and grape seed extracts (standardized to hydroxytyrosol and procyanidins, respectively) to both 10‐week‐old male C57/BL mice and 15‐week‐old female New Zealand white rabbits before surgically induced osteoarthritis. Compared with untreated controls, animals receiving the combined extracts had reduced cartilage damage and preserved aggrecan expression in the cartilage. The combined treatment also counteracted the effects of IL‐1*β* on primary cultured rabbit chondrocytes, further demonstrating its anti‐inflammatory and chondroprotective potential [84]. In a posttraumatic osteoarthritis model induced by anterior cruciate ligament transection in rats, a diet supplemented with extra‐virgin olive oil combined with treadmill exercise restored lubricin, a cartilage‐derived glycoprotein, and normalized IL‐1 levels in cartilage and synovial fluid. After 8 weeks of experiment, animals receiving both interventions showed values comparable to healthy controls [85].

Although limited in number, clinical trials with olive oil or its derivatives have also demonstrated outcomes in osteoarthritis consistent with those observed in in vivo models (Table 1). In a randomized, double‐blinded, placebo‐controlled trial, Bitler et al. [6] examined the effects of an oral supplement with 400 mg of freeze‐dried olive water extract daily for 8 weeks in 30 patients with osteoarthritis (aged 55–75 years). This regimen resulted in a significant reduction in disability according to the Health Assessment Questionnaire‐Disability Index, together with an improvement in disease activity as reflected by the Disease Activity Score with 28‐Joint Count Index [6]. In a small cohort of participants with knee osteoarthritis, the administration of an oral hydroxytyrosol supplement for 4 weeks (50.1 mg/day of olive extract providing 10.04 mg hydroxytyrosol) was investigated in a randomized, double‐blinded, placebo‐controlled setting. The intervention resulted in a more evident overall clinical improvement than placebo, as indicated by the Japanese Orthopaedic Association Score. The most consistent benefit concerned pain experienced during sleep, with a slight improvement also observed in pain experienced while walking on a flat surface, whereas the other pain‐related outcomes did not differ substantially from placebo [7].

**Table 1:** Summary of clinical trials on olive oil and its derivatives in osteoarthritis.

Study and reference	Design	Participants	Intervention	Duration	Main outcomes	Key findings	Limitations
Bohlooli et al., 2012 [5]	Randomized, double‐blinded, controlled trial	60 female patients with OA (40–85 years)	1 g gel with virgin olive oil (topical) vs. 0.5% Piroxicam gel, three times daily	4 weeks	WOMAC score, secondary outcomes (pain, function)	Improvement in pain from second week	High dropout rate, relatively small sample size, short trial duration
Bitler et al., 2007 [6]	Randomized, double‐blinded, placebo‐controlled trial	63 patients with OA (55–75 years)	400 mg freeze‐dried olive water extract vs. placebo, daily	8 weeks	HAQ‐DI, DAS‐28	Improvements in disability and disease activity	Relatively small sample size, short trial duration
Gelmini et al., 2016 [8]	Time‐series study	5 patients with knee and hand OA (mean age: 60.2 ± 8.1 years)	5 g ointment (5% olive oil unsaponifiable fraction), three times daily	3 weeks	Pain, edema, redness, heat, and mobility	Improvement in pain, edema, redness, heat and mobility within 1–2 weeks	Very small sample size, short trial duration, no blinding, no placebo‐controlled
Takeda et al., 2013 [7]	Randomized, double‐blinded, placebo‐controlled trial	25 patients with knee OA (mean age: 60.8 ± 7.2)	50.1 mg olive extract (10.04 mg hydroxytyrosol) vs. placebo daily	4 weeks	Japanese Orthopaedic Association Scores	Reduced pain during sleep, marginal improvement in walking pain on a flat surface	Very small sample size, short trial duration, limited outcomes

Abbreviations: DAS‐28, disease activity score with 28‐joint count; HAQ‐DI, health assessment questionnaire‐disability index; OA, osteoarthritis; UF, unsaponifiable fraction; WOMAC, Western Ontario and McMaster Universities Osteoarthritis Index.

In two other studies, olive oil or its bioactive compounds were administered topically. A first study utilized topical olive oil in a double‐blinded, randomized clinical trial involving female Iranian patients with osteoarthritis (aged 40–85 years). In this trial, participants were assigned to either 0.5% piroxicam gel (*n* = 30) or topical virgin olive oil (*n* = 30), each applied three times daily for 4 weeks. Both groups experienced reductions in the Western Ontario and McMaster Universities Osteoarthritis Index (WOMAC) pain subscale, although the olive oil group showed more pronounced improvements beginning in the second week [5]. Similarly, Gelmini et al. explored in a preliminary study the use, three times daily for 3 weeks, of a topical ointment containing 5% unsaponifiablefrom unripe olive fruits in five individuals with symptomatic osteoarthritis (mean age: 60.2 ± 8.1 years). Within 1 week of treatment, subjects reported reductions in inflammation‐induced joint pain and edema, as well as improved mobility, while redness and heat showed improvement by 2 weeks [8].

## 5. Anti‐Inflammatory and Senotherapeutic Properties of Olive Oil Bioactive Compounds Relevant to Osteoarthritis

In the following paragraphs, we will explore in more detail the key aspects and molecular pathways through which various bioactive compounds of olive oil may synergistically exert their anti‐inflammatory and senotherapeutic activities. Many of these compounds have been investigated in in vitro or in vivo models of osteoarthritis and are reported in Table 2.

**Table 2:** Olive oil–derived compounds investigated in osteoarthritis models (in vitro or in vivo).

Olive oil compounds	Study model	Key mechanisms	Effects in osteoarthritis	References
Hydroxytyrosol	In vitro (primary human chondrocytes, C‐28/I2 cells)	‐ Reduction of H_2_O_2_‐induced ROS and DNA breakage ‐ Inhibition of caspase‐3 ‐ Downregulation of COX2, iNOS, MMP13 ‐ Enhancement of SIRT1 expression	‐ Protection against oxidative damage and apoptosis ‐ Attenuation of inflammatory markers and cell senescence features	[86, 87]
Ligstroside aglycone	In vitro (human osteoarthritis cartilage explants)	‐ Downregulation of proinflammatory genes (NOS2 and MMP13)	‐ Alleviation of proteoglycan degradation ‐ Suppression of pro‐inflammatory factors	[88]
Oleic acid	In vitro (chondrocytes)	‐ Inhibition of autophagy signaling ‐ Increased NOX4 expression and ROS levels	‐ Pro‐apoptotic effects in chondrocytes ‐ Promotion of ROS production and oxidative damage	[89, 90]
Oleocanthal	In vitro (ATDC‐5 chondrocytes)	‐ Decrease in iNOS expression ‐ Decrease in IL‐1*β*, TNF‐*α*, and GM‐CSF	‐ Reduced NO production and inflammatory mediator release	[91, 92]
Oleuropein	In vivo (Dunkin–Hartley guinea pig model); In vitro (osteoarthritic chondrocytes, mesenchymal stem cells, synovial cells, bone cells)	‐ Decreased PGE2 and collagen‐2 catabolism ‐ Reduction of NF‐*κ*B activity (IL‐1*β*, IL‐6, COX2, and MMP3) ‐ Reduction of Cx43 and Twist‐1 activity	‐ Reduced histological scores and inflammation ‐ Improvements in cartilage integrity and chondrocyte redifferentiation ‐ Decreased senescence markers and SASP factors in joint‐related cells	[4, 93]

Abbreviations: ATDC‐5, murine chondroprogenitor cell line; C‐28/I2, human immortalized chondrocyte cell line; COX2, cyclooxygenase‐2; Cx43, connexin43; GM‐CSF, granulocyte‐macrophage colony‐stimulating factor; IL, interleukin; iNOS, inducible nitric oxide synthase; MAPK, mitogen‐activated protein kinase; MMP, matrix metalloproteinase; NF‐*κ*B, nuclear factor *κ*B; NO, nitric oxide; NOX4, NADPH oxidase 4; PGE2, prostaglandin E2; ROS, reactive oxygen species; SASP, senescence‐associated secretory phenotype; SIRT1, sirtuin 1; TNF‐α, tumor necrosis factor α.

### 5.1. Oleic Acid and Omega‐3 PUFA

Oleic acid represents the major monounsaturated component of the olive oil saponifiable fraction. Experimental data demonstrate that oleic acid may lower levels of inflammatory cytokines (TNF‐*α*, IL‐6, and IL‐1*β*). Molecular docking studies suggest that oleic acid interacts with Toll‐like receptors (TLRs)3 and TLR4, forming ligand‐protein complexes with high predicted binding affinity. These receptors play a pivotal role in both innate and adaptive immunity and facilitate the nuclear translocation of NF‐*κ*B. Additionally, oleic acid suppresses the expression of key components of the MAPK pathway, such as JNK and p38 MAPK, as well as the NF‐*κ*B pathway, including I*κ*B, COX2, and PGE2. In vivo findings reveal that oleic acid reduces inflammatory cell counts and decreases cytokine levels (IL‐4, IL‐6, and TNF‐*α*) [94–98]. However, it is well known that oleic acid, either alone or in combination with other fatty acids, demonstrates proapoptotic potential in chondrocytes by inhibiting autophagy signaling, enhancing NADPH oxidase 4 (NOX4) expression, and consequently increasing ROS production, thus promoting osteoarthritis progression [89, 90].

Omega‐3 PUFA, although present in smaller concentrations compared to oleic acid, may have a role in modulating inflammation. Omega‐3 PUFA serve as precursors for the synthesis of specialized proresolving mediators (SPMs), such as resolvins, protectins, and maresins, which actively contribute to the resolution of inflammation and tissue homeostasis. In contrast, omega‐6 PUFA may promote the production of proinflammatory eicosanoids, such as prostaglandins and leukotrienes, which are involved in the initiation and amplification of inflammatory responses. The balance between omega‐3 and omega‐6 fatty acids may be therefore critical in determining the overall inflammatory state, influencing processes such as immune regulation, oxidative stress, and chronic disease progression [99, 100]. Although the results of the trials performed to date are not consistent, the majority indicate a beneficial effect of omega‐3 PUFA on telomere length counteracting senescence [101].

### 5.2. Ligstroside and Oleocanthal

Although unsaponifiable represents a small proportion, it is rich in compounds with evidence of anti‐inflammatory and anti‐senescence activity. Ligstroside aglycon exhibited significant antioxidant and anti‐inflammatory effects by reducing oxidative stress markers (NO, iNOS, NOX‐1) and downregulating proinflammatory cytokines, COX2, and microsomal prostaglandin E synthase‐1 (mPGEs‐1) in lipopolysaccharide (LPS)‐stimulated murine peritoneal macrophages. Its protective mechanisms involve activating one of the main antioxidant pathways, the nuclear factor erythroid 2‐related factor 2 (Nrf2)/heme oxygenase 1 (HO‐1) pathway, while inhibiting the signaling of NF‐*κ*B, MAPKs, and Janus kinase 2 (JAK2)/STAT3. This last is involved in the initiation and progression of inflammatory responses. Additionally, ligstroside aglycon suppressed both canonical and noncanonical NLRP3 inflammasome activation, a cytosolic multiprotein complex of the innate immune system responsible for the activation of inflammatory responses [102]. Similarly, a chemically modified ligstroside aglycon markedly downregulated the expression of proinflammatory genes, including *NOS2* and *MMP13*, and reduced NO release and proteoglycan degradation in human osteoarthritis cartilage explants [88]. In a recent study, ligstroside aglycon and oleocanthal showed beneficial effects on mitochondrial respiration and dysfunction. This aspect is directly linked to senescence, given that a reduction in respiratory capacity per mitochondrion, along with a decreased mitochondrial membrane potential, typically accompanied by increased production of ROS, serves both as a cause and a consequence of cellular senescence [103].

Precisely, oleocanthal has attracted significant attention due to its similarities to nonsteroidal anti‐inflammatory drugs, particularly for its effects on macrophages and chondrocytes and its action as a TLR4 antagonist and activator of other potential anti‐inflammatory pathways [104]. Macrophages, as key sources of inflammatory cytokines, have been a primary focus in studying the anti‐inflammatory properties of oleocanthal. In LPS‐stimulated J774 murine macrophages, oleocanthal was found to suppress NO production and reduce the expression of inflammatory cytokines, including IL‐1*β*, TNF‐*α*, and granulocyte‐macrophage colony‐stimulating factor (GM‐CSF). Additionally, it downregulated both the protein and gene expression of macrophage inflammatory protein 1*α* (MIP‐1*α*) and IL‐6 in macrophages and ATDC5 chondrocytes [92]. Similarly, oleocanthal has been shown to protect ATDC5 chondrocytes from LPS‐induced cell death at low concentrations (1–10 µM). This protection is largely attributed to its ability to reduce iNOS expression, thereby lowering NO production [91].

### 5.3. Oleuropein

Oleuropein has been studied for its anti‐inflammatory and chondroprotective effects. In an interesting study, Dunkin–Hartley guinea pigs, which develop spontaneous osteoarthritis, were supplemented with oleuropein, rutin, or a combination of rutin and curcumin for 31 weeks. Histological evaluation indicated that oleuropein supplementation was associated with an attenuation of joint lesion severity, a reduction in cellularity scores, and less pronounced synovial inflammation. Consistent with these findings, biochemical analyses showed lower levels of PGE_2_ and reduced collagen‐2 degradation, supporting a protective effect of oleuropein against osteoarthritis‐related inflammatory and cartilage damaging processes [93]. There are different molecular mechanisms by which oleuropein and its derivatives (oleacin and oleuropein aglycone) confer their beneficial effects. These compounds have been shown to reduce production of proinflammatory cytokines and adhesion molecules in LPS‐stimulated monocyte/macrophages (THP‐1) and to decrease proinflammatory cytokine levels and NO production, by downregulating iNOS expression, in LPS‐stimulated RAW 264.7 murine macrophages. In this last model, they also increase the expression of anti‐inflammatory genes and the production of anti‐inflammatory cytokines (IL‐10 and TGF‐*β*) [105, 106]. A recent study investigated the effects of oleuropein on key factors in osteoarthritis, specifically the overactivity of the gap junction protein connexin43 (Cx43) and the accumulation of senescent cells, both of which are associated with impaired tissue regeneration. The results indicate that oleuropein modulates Cx43 promoter activity and promotes the differentiation of human mesenchymal stem cells into chondrocytes and osteoblasts while reducing adipogenesis. In osteoarthritic chondrocytes, oleuropein decreases Cx43 levels and Twist‐1 activity, facilitating redifferentiation, and restoring the synthesis of essential cartilage ECM components, including collagen type II *α*1 chain (Col2A1) and proteoglycans, and reducing SASP mediated by NF‐*κ*B (IL‐1*β*, IL‐6, COX2, and MMP3). Moreover, oleuropein lowers cellular senescence in osteoarthritic chondrocytes as well as in synovial and bone cells [4].

### 5.4. Hydroxytyrosol and Other Phenolic Compounds

Hydroxytyrosol shows the ability to modulate specific signaling pathways in chondrocytes. This phenolic compound reduces hydrogen peroxide (H_2_O_2_)‐induced ROS production and associated DNA damage in primary human chondrocytes, preventing H_2_O_2_‐driven chondrocyte apoptosis by inhibiting caspase‐3 activation. Hydroxytyrosol also attenuates the effects of the growth‐related oncogene *α* (GRO*α*), a chemokine involved in the pathogenesis of osteoarthritis, reducing crucial mediators of inflammation, cartilage degradation, and terminal chondrocyte differentiation. Furthermore, GRO*α*‐induced downregulation of gene *SIRT1*, which is associated with osteoarthritis, is reversed by hydroxytyrosol treatment [86]. Similarly, hydroxytyrosol may enhance SIRT1 expression in both primary human chondrocytes and C‐28/I2 chondrocyte cell lines [87]. The senotherapeutic properties of hydroxytyrosol are also linked to the activation of the AMPK pathway and autophagy through sirtuin 1 (SIRT1)‐dependent and ‐independent mechanisms [107, 108]. Recently, it was demonstrated that hydroxytyrosol inhibited the SASP with a reduction in a concentration‐dependent manner of IL‐1*β* and IL‐6 release in TNF‐*α*‐stimulated chondrocytes. In addition, hydroxytyrosol promoted cell autophagy through the increase of SIRT6 mRNA and protein levels [109].

Although not directly investigated in osteoarthritis, other phenolic compounds have demonstrated anti‐inflammatory and senotherapeutic effects in different disease models. Tyrosol (and its metabolites) exerts anti‐inflammatory action through the inhibition of the NF‐*κ*B pathway, the reduction of proinflammatory factors and adhesion‐related molecules, such as C‐reactive protein, TNF‐*α*, IL‐6, monocyte chemotactic protein‐1 (MCP‐1), intercellular adhesion molecule‐1 (ICAM‐1) and vascular cell adhesion molecule‐1 (VCAM‐1) [110–113]. Similarly, the lignan pinoresinol inhibits the inflammatory responses in LPS‐activated primary microglia, blocking the production of NO, PGE_2_, TNF‐*α*, IL‐1*β*, and IL‐6, and reducing mRNA and protein levels of iNOS and COX2 principally through the inhibition of the NF‐*κ*B pathway [114].

Caffeic acid reduced SASP in rheumatoid arthritis‐derived fibroblast‐like synoviocytes reducing the production of IL‐6 and TNF‐*α*, and repressing PGE_2_ and MMP1 by inhibiting phosphorylation of NF‐*κ*B inhibitor kinase (I*κ*B kinase/IKK) *α*/*β* and I*κ*B&*α* [115]. The flavonoid apigenin also reduced the SASP in part by inhibiting IL‐1*α* signaling principally through p38‐MAPK and NF‐*κ*B. Notably, apigenin demonstrated particular efficacy in reducing the expression and secretion of C‐X‐C motif chemokine ligand 10 (CXCL10), a recently identified SASP factor [116]. Luteolin, another flavonoid present in olive oil, beyond its pleiotropic anti‐inflammatory effects, reduces proinflammatory SASP factors and modulates multiple signaling pathways implicated in cellular senescence, including NF‐*κ*B, JAK/STAT, and TLRs [117, 118].

### 5.5. Other Bioactive Compounds

Various other bioactive compounds in olive oil, including squalene, phytosterols (*β*‐sitosterol, *Δ*5‐avenasterol, campesterol, and stigmasterol), vitamins (particularly vitamin E), and carotenoids (*β*‐carotene, lutein, violaxanthin, and neoxanthin), are recognized for their anti‐inflammatory and senotherapeutic effects. Collectively, these olive oil‐derived compounds target multiple signaling pathways, including NF‐*κ*B, MAPK, JAK/STAT, and TLRs. For example, squalene exerts its anti‐inflammatory activity in part by modulating NF‐*κ*B signaling, thereby reducing the production of proinflammatory cytokines and enzymes such as IL‐6, TNF‐*α*, iNOS, and COX2. In addition, squalene has been reported to inhibit oxidative stress by enhancing the activity of antioxidant enzymes, a mechanism that can indirectly diminish cellular senescence [119, 120].

Phytosterols, in particular, *β*‐sitosterol, campesterol, and stigmasterol have demonstrated notable immunomodulatory and anti‐inflammatory properties. These molecules can mitigate inflammation through attenuation of NF‐*κ*B and NLRP3 via AMPK, ultimately lowering the expression of various proinflammatory mediators. By suppressing chronic, low‐grade inflammation, phytosterols also contribute to reducing cellular senescence, which is often triggered by persistent inflammatory stimuli [121–123]. Furthermore, some sterols interact with TLRs, further dampening inflammatory signaling cascades [124]. This multifaceted modulation of immune responses has significant implications for preventing the SASP that drives tissue dysfunction and aging.

Vitamin E, present in trace amounts in olive oil, exerts anti‐inflammatory effects through the regulation of both innate and adaptive immune responses, largely by influencing the NF‐*κ*B pathway and NLRP3‐mediated inflammatory processes [125–127]. By promoting adequate telomere length and mitigating SASP, vitamin E may indirectly alleviate the accumulation of senescent cells [128, 129].

Carotenoids, including *β*‐carotene, lutein, violaxanthin, and neoxanthin, function as potent antioxidants by scavenging ROS and protecting cellular components from oxidative injury [69]. Persistent ROS levels are a key factor driving both inflammation and senescence. Carotenoids, in particular *β*‐carotene, may reduce the activation of redox‐sensitive transcription factors including NF‐*κ*B, STAT3, and activator protein‐1 (AP‐1), thus lowering proinflammatory gene expression. Their action in enhancing cellular antioxidant defenses not only helps prevent oxidative damage but also may diminish the stress‐induced senescence [130].

## 6. Conclusion

Osteoarthritis is increasingly recognized as a multifactorial disease in which chronic inflammation and cellular senescence act synergistically to promote joint degeneration. The inflammatory cascade, triggered by tissue damage and sustained by NF‐*κ*B and MAPK activation, induces chondrocyte senescence and the secretion of proinflammatory mediators that perpetuate a self‐amplifying cycle of inflammation and matrix degradation.

Bioactive compounds derived from olive oil exhibit promising potential in modulating these processes by attenuating oxidative stress, inhibiting SASP factors, and restoring cellular homeostasis. Their combined effects on multiple signaling pathways, including NF‐*κ*B, MAPK, and JAK/STAT, suggest a multifaceted protective action capable of interrupting the crosstalk between inflammation and senescence.

However, the current pharmacokinetics/pharmacodynamics properties and clinical evidence remain limited and dated. The few available trials, often limited by small sample sizes and methodological constraints, were conducted more than a decade ago, and no substantial clinical progress has been achieved since then. This highlights a significant research gap and the need for renewed clinical attention. Future studies should aim to validate preclinical findings through large, well‐designed randomized trials, clarify dose–response relationships and bioavailability, and assess the long‐term safety of olive oil bioactives, together with a clearer understanding of their potential interactions with conventional pharmacological therapies in osteoarthritis. Integrating modern *omics*, chemometrics, imaging, and biomarker‐based approaches will be essential to better characterize the bioactive metabolites and individual responses and to develop targeted nutraceutical strategies for osteoarthritis prevention and management.

## Author Contributions

Alessandro Medoro, Roberta Tardugno, and Sergio Davinelli contributed to writing—original draft. Maria Lisa Clodoveo, Eugenio Luigi Iorio, and Magda Tsolaki contributed to writing—review and editing. Giovanni Scapagnini and Filomena Corbo contributed to conceptualization, review, and editing. All authors have read and agreed to the published version of the manuscript.

## Funding

This research was funded by the F.F.I.N—Functional Foods Italy Network (POS TR5—Piano Operativo Salute Traiettoria 5) of the Italian Ministry of Health (Grant CUP H33C22000780008).

## Conflicts of Interest

The authors declare no conflicts of interest.

## Data Availability

Data availability is not applicable to this article as no new data were created or analyzed in this study.
